# Spatio-temporal migratory dynamics of *Jasus frontalis* (Milne Edwards, 1837) in Alexander Selkirk Island, Juan Fernández archipelago, Chile

**DOI:** 10.1371/journal.pone.0200146

**Published:** 2018-07-25

**Authors:** Catalina Román, Billy Ernst, Martin Thiel, Pablo Manríquez, Julio Chamorro

**Affiliations:** 1 Programa de Magíster en Ciencias con mención en Pesquerías, Universidad de Concepción, Concepción, Chile; 2 Instituto de Fomento Pesquero, Valparaíso, Chile; 3 Departamento de Oceanografía, Facultad de Ciencias Naturales y Oceanográficas, Universidad de Concepción, Concepción, Chile; 4 Núcleo Milenio INVASAL, Concepción, Chile; 5 Facultad de Ciencias del Mar, Universidad Católica del Norte, Coquimbo, Chile; 6 Millennium Nucleus Ecology and Sustainable Management of Oceanic Island (ESMOI), Coquimbo, Chile; 7 Centro de Estudios Avanzados en Zonas Áridas (CEAZA), Coquimbo, Chile; University of Minnesota, UNITED STATES

## Abstract

Knowledge about the spatial patterns and movements of crustaceans has gained importance since the creation of marine protected areas and the development of spatial management for benthic ecosystems. The Juan Fernández spiny lobster (*Jasus frontalis*) is an endemic marine species and most valuable resource that exhibits migratory dynamics in a highly spatially regulated fishery. To study movement patterns around Alexander Selkirk Island, a mark-recapture program was implemented in 2008, when approximately 7000 non-commercial (undersized) lobsters were tagged and followed for nearly 14 months. Using quantitative georeferenced data, this study revealed spatial structuring of Juan Fernández spiny lobster and tested hypotheses about alongshore and inshore-offshore movements. Eight clusters were identified around Alexander Selkirk Island, with moderate time-varying connectivity between them. Seasonal inshore-offshore movements were detected all around the island, but more conspicuously to the north. Average travelling distance was 1.2 km (1.7 sd). Our results confirmed that towards the end of austral spring males and females embark in a seasonal offshore migration to deeper waters, returning to shallower waters only during winter. These findings quantitatively consolidate the conceptual migratory model that local fishermen had already inferred for this resource from about a century of sustainable fishing.

## Introduction

The distribution and movements of benthic marine resources constitute important aspects of population dynamic studies [1]. Aggregation, territorial behavior, and migratory patterns are features commonly known by fishers worldwide, but the underlying processes often remain unresolved by scientists [2–4]. The advent of Marine Protected Areas (MPAs) and spatial regulations of marine fisheries have prompted the need to know the movement patterns and distances of exploited resources [5–8].

In several large crustacean species, especially decapods, migrations have been well studied [9]. Herrnkind [10] reviewed movement patterns of palinurid lobsters and described three mayor types: *migration*, wherein an individual or population moves a considerable distance often (but not always) periodically or with a return to the original area; *nomadism*, by individuals over a large area without clear-cut start and end points; and *homing*, involving periodic (often daily) movements from a shelter to some nearby area with subsequent return to that shelter or others nearby.

In palinuroid species, there is indication that females migrate away from coastal reefs and banks to release larvae into oceanic currents [10], then returning to the native foraging-sheltering grounds [11]. Seasonal inshore-offshore migration in *Jasus edwardsii* appears to be associated with molting and reproduction, and can exceed distances of 5 km. In southern New Zealand [12] large numbers of female lobsters migrate inshore during autumn to molt with mating and egg extrusion a few months later. Large males are found inshore to molt in late austral spring. For *Panulirus ornatus*, seasonal movements have been reported across the Gulf of Papua to release larvae in areas where oceanic currents are favorable to larval dispersion. During migration, the ovary is developed and larval release occurs during austral spring-summer once lobsters entered the reef system [13]. In clawed lobsters (Nephropidae) migratory patterns have been documented for *Homarus americanus*; in New England, movement was inshore in spring and summer returning offshore in fall and winter. This movement is presumably promoted by the high temperatures in shallow waters during the summer season, which could favor reproductive processes such as molting, mating and spawning in females. Return migrations in the winter season to deeper waters may be an escape from the wave turbulence and cold water temperatures that are frequent in shallow waters during the winter season [14]; in southern Gulf of Maine, ovigerous lobsters tracked with ultrasonic telemetry showed a movement pattern to offshore areas during winter, where they appeared to remain until after their eggs hatched in early summer [15]. In the family Scyllaridae, there is evidence of a seasonal pattern of adult lobsters *Scyllarides latus* that move to deeper-cold waters in mid-summer, triggered by the high temperatures (28°C-29°C) registered in coastal regions [16,17] Brachyuran crabs also exhibit seasonal and reproductive migrations [9]; *Chionoecetes opilio* undergo an ontogenetic migration in which older individuals move to deeper waters and return to shallow waters to mate with older multiparous females. Females start their movement after the puberty molt and primiparous mating occurs during boreal winter and hatching takes place after a year. Distances moved by females can reach 100 km, moving offshore during boreal winter and inshore during boreal spring-summer [18,19]. While the general movements are relatively well known in many large crustaceans, there are some others in which this process is only poorly understood, especially the seasonal movement patterns.

**Fig 1:**
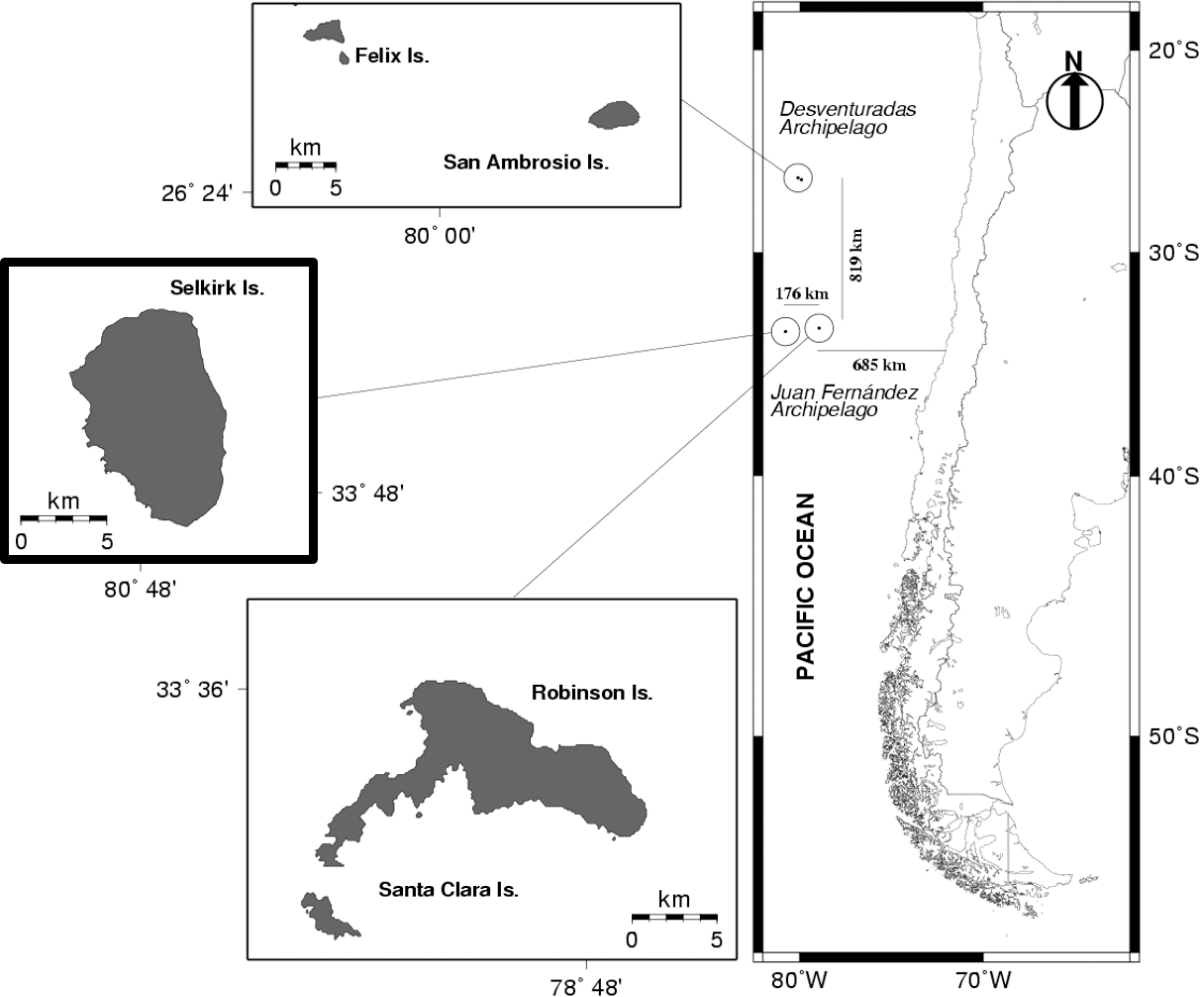
Geographic distribution of the Juan Fernandez spiny lobster (*Jasus frontalis*) fishery. Box with thick border indicates the study area.

*Jasus frontalis*, a palinurid achelate lobster, is an endemic resource and the main economic driver of Juan Fernández Archipelago (JFA, Fig 1) [20,21]. This spiny lobster is distributed over the insular shelf around the islands up to 180 meters deep and is fished seasonally (October to May) by a small fully integrated community of artisanal fishermen [22,23]. The fishery has been internationally recognized by its sustainable practices, obtaining in 2015 the MSC certification (https://www.msc.org/). The spatial component plays a key role to this fishery because access to the resource has long been regulated by an informal but well-structured traditional sea tenure system [21]. Fishermen set their traps at individual discrete fishing spots called *Marcas* [22], shifting locations and depths during the course of the fishing season; initially placing them near the coast with a subsequent move to deeper waters [23,24]. These shifts are perceived by fishermen as population movement between shallow and deeper waters throughout the fishing season, as occurs for large decapod species around the world [25]. The spatial distribution of the *Marcas* and the temporal component associated with their use suggest a conservative clustering pattern maintained across season [21]. Despite the relevance of this fishery for human well-being, basic aspects of its spatial structure and movement patterns still remain little known.

In this contribution, we investigate the spatial structure of *J*. *frontalis* around Alexander Selkirk Island using georeferenced data on individual discrete fishing spots and we analyzed its alongshore and inshore-offshore migration using recapture data from an intensive biological sampling program. This work presents evidence of cluster structures, movement between clusters and coastal-ocean migrations.

### Brief overview of the system and the fishery around JFA

The JFA system is located in the Southeastern Pacific Ocean and belongs to a submarine ridge of ~425 km length [26]. The archipelago comprises three islands: Robinson Crusoe (RC; 47.9 km^2^), Santa Clara (SC; 2.2 km^2^) and Alexander Selkirk (AS; 49.5 km^2^), which are recognized for hosting high diversity and endemism [27,28]. AS Island (33°45'S, 80°45'W) is located 800 km west of the South American continent and is the most westerly island in the JFA [29]. It is described by visitors as a rugged, desert dome-shaped mountain with high contours and slopes interrupted by gorges that reach 1300 meters (Fig 1). The island has a small marine shelf that contains sandy and muddy bottoms, temperate rocky reefs and a rich diversity of endemic neritic and benthic species [30]. Oceanographic influences over the geomorphological and climate conditions [31] make this island an inaccessible territory, which is only inhabited by a small community of fishermen and their families during the fishing season [32].

The size of the fleet in AS Island increased from 10 (historically) to 14 boats in recent seasons. The fleet is composed of double-ended wooden boats of 8–10 m length and also some fiberglass boats [33,34]. Gear consists of rectangular wooden traps (1.2–1.3 m length; 0.4 m height; 0.7–0.8 m width) where bait (small pelagic, whitefish and moray eels) is placed. When weather permits the fishermen go out every day during the fishing season; individual traps are serviced every other day, with an average of 41 traps deployed per trip [21]. Commercial catch per unit effort is around 1.5 lobsters per trap and for non-retained lobsters is on average 23.5 individuals per trap [35]. Formal regulations on the harvest have been of the “SSS” strategy type: a legal-size limit (115 mm carapace length), a closed season (May 15–Sept 30), and a prohibition on harvesting egg-carrying females [36]. Additionally, there is an informal “*Marcas*” sea tenure system that consists of individual discrete fishing spots (“owned” by individual fishermen or members of their families) where traps are deployed. They are traditionally identified by alignments of land features or more recently by GPS waypoints. A previous contribution has identified about 1000 *Marcas* for AS Island [21] organized into 85 toponyms, sector names used by fishermen to assist the geolocation of their *Marcas*. During the fishing season, a fisherman might relocate a trap in close vicinity of a *Marca* or use different *Marcas* from his pool of *Marcas* (e.g. nearshore and offshore) [21,22]. Larger-scale clusters of *Marcas* around the island that could potentially be associated to ecological units have not been previously described for AS island.

## Materials and methods

This study was not carried out on private lands. No specific permission was required because fisheries activity is submitted to national waters and the Undersecretary of Fisheries provide permissions to carried out this study. In this study, no endangered or protected species were injured.

### Mark-recapture study

**Fig 2:**
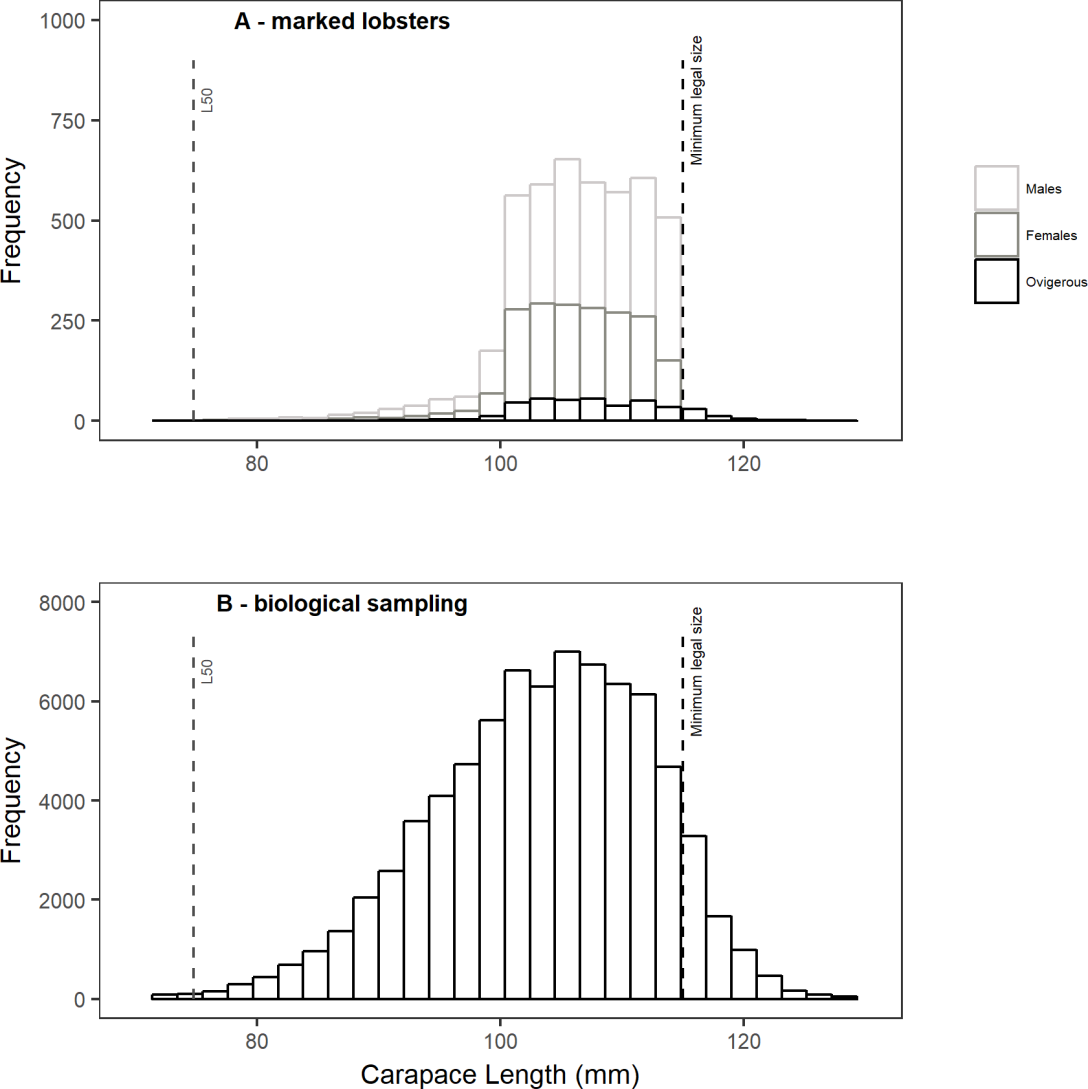
Length frequency distribution of tagged lobsters. (A) Marked lobsters by sex for all tagging periods and (B) Size structure (sex combined) from biological sampling during 2008/09 fishing season [35]. Vertical dashed lines correspond to size of 50% of maturity and minimum legal size.

During the 2008/09 fishing season, a mark-recapture program was implemented to obtain information on spatio-temporal movement patterns and demographic rates (this research was carried out by the University of Concepción (Chile) through a research grant from the Undersecretariat of Fisheries, Chile). T-bar tags were used (FD-68B, made by Floy Tag, Seattle USA) and applied with a MARK II grip gun. Tags were applied dorsally, between the thorax and the first abdominal segment, at intramuscular level to avoid losses during the moult. A total of 6895 non-damaged lobsters were effectively tagged. After recording temporal/spatial and biological information (size, sex), lobsters were immediately released at their location of capture. Size of tagged lobsters comprised a main group between 100 and 115 mm CL, mostly undersized lobsters (Fig 2A) that were not removed by the fishery until they reach 115 mm CL. This size range is representative of the sizes observed regularly in biological samplings [37] (Fig 2B).

Taking into account the multi-objective nature of the tagging program, namely movement characterization, survival rates and somatic growth [35], and previous knowledge about this stock and its fishery, the tagging was conducted in three different periods from October 2008 to April 2009 (Table 1). The first tagging event (M1; 3093 lobsters marked) occurred in early October 2008, at the onset of the 2008/09 fishing season, when the fishery is mainly operating inshore and the male moult (January [37]). The second tagging event (M2; 1712 lobsters marked) was developed from mid-February to mid-March, right after the austral summer male moult and when the fleet reached its offshore distribution. A third tagging (M3; 2090 lobsters marked) occurred in April 2009, right before the austral winter fishery closure.

**Table 1:** Spatio-temporal stratification of mark and recapture periods for movement analysis across 2008/2009 fishing season.

	2008			2009										
	Oct	Nov	Dec	Jan	Feb	Mar	Apr	May	Jun	Jul	Aug	Sep	Oct	Nov
Mark	M1				M2		M3							
Recapture periods	R1	R1	R1	R2	R2	R2	R2						R3	R3
Main fishing operation area	Inshore	Inshore	Inshore	Offshore	Offshore	Offshore	Offshore	*	*	*	*	*	Inshore	Inshore

*: Winter fishing closure

### Movement analysis inputs

To analyze movement, temporal and spatial variables were considered. Also, results on connectivity levels around the island were used to structure the inshore-offshore modelling.

#### Temporal stratification

**Fig 3:**
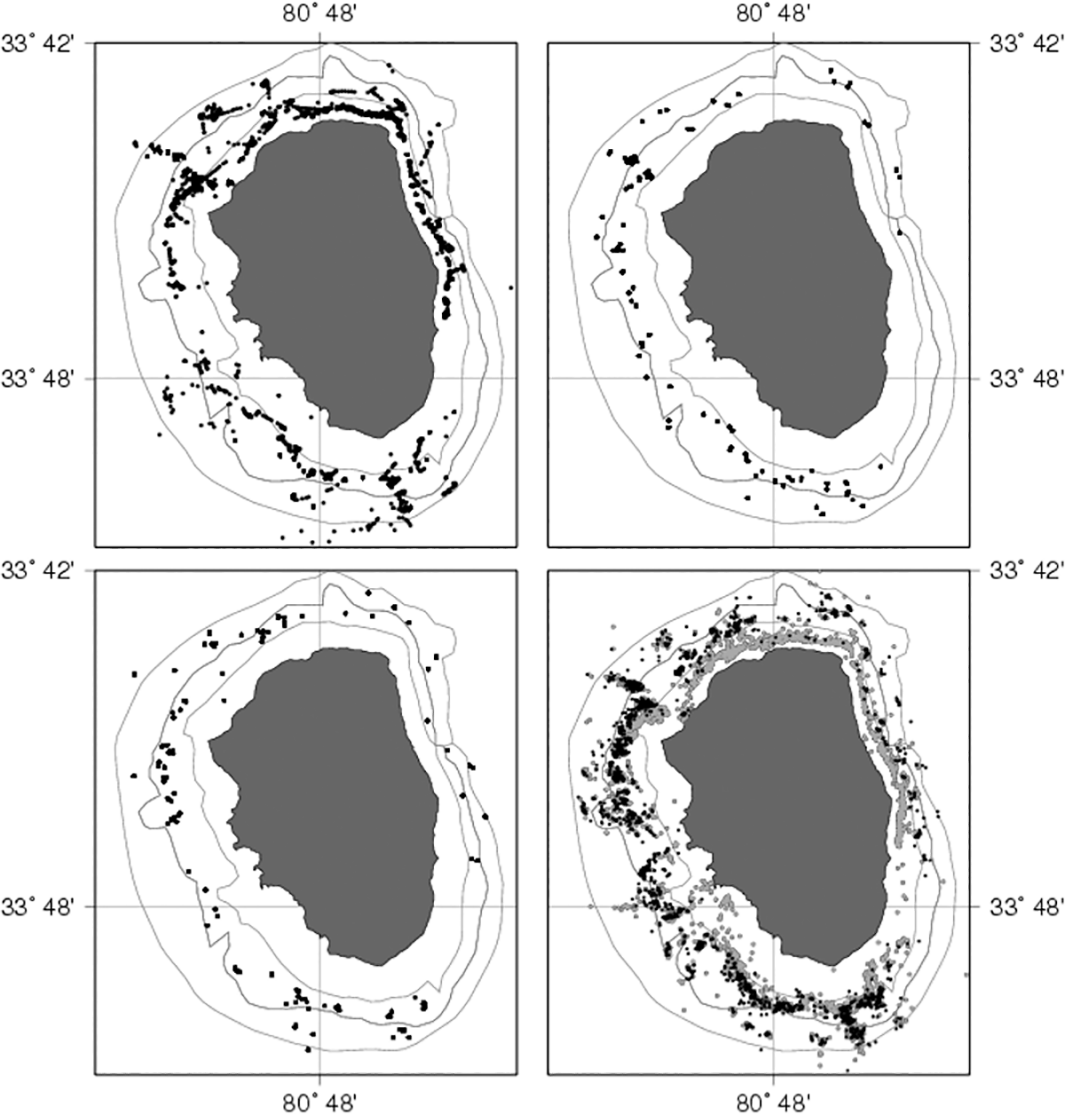
Spatial location of the marking events around AS Island and the geographic points where traps were regularly deployed throughout the fishing season. Marking events from October 2008 (M1, upper left box), February-March 2009 (M2, upper right box), April 2009 (M3, lower left box) and all sampling points (obtained from fishing effort) around AS Island from October 2008 to December 2009 (lower right box).

Considering the marking events shown in Fig 3 and previous work on which this figure was based [21–24], recapture information was analyzed by month of recapture ordered in 3 different recapture periods: (i) a first recapture period (R1; October to December, 2008), during the first part of the fishing season and before fishermen move their traps from shallow to deeper waters, (ii) the second part of the 2008/09 fishing season (R2; January to April, 2009), and (iii) a third recapture period (R3; October and November 2009) representing the onset of the 2009/10 fishing season (Table 1). In this sense, for lobsters marked in M1 (first marking event), there are three recapture periods (M1R1, M1R2 and M1R3); for the second marking event there are two recapture periods (M2R2 and M2R3) and for the third marking event only one recapture period (M3R3).

The spatial distribution of our capture/recapture effort was conditioned by the distribution of the fishing fleet. In order to test if sampling effort distribution on a bathymetric axis was unbalanced, we conducted a proportion-based Chi-squared test for different time periods and bathymetric strata.

#### Spatial stratification

Due to the inexistent bathymetry database for this island, we created a topographic model in ArcGIS using two pieces of information, namely echo-sounding transects recorded during opportunistic fishing trips (Ernst, unpublished data) and depth records obtained from the routine biological sampling of the lobster monitoring program, registered in logbooks. Three depth strata, based on the topographic model, were created: b1—shallow (0–50 meters), b2—intermediate (51–100 meters), and b3—deep level (>101 meters). Considering the shape of the island [29] and that shallow bathymetry is associated with coastal areas, the first level b1 is considering an inshore area, while b2 and b3 levels are considered offshore areas.

Identification of zones around AS Island was made by cluster classification analysis, using Gaussian mixture models. This analysis was aimed at identifying non-arbitrary strata to study lobster movements, as opposed to using the high number of fishing toponyms (85 sectors) described for AS Island or the arbitrary statistical zones used in the monitoring program of the fishery (6 zones). Model-based method classification [38] provided an objective statistical approach to clustering considering multivariate normality; this assumption together with previous information of individuals helps in the definition of resultant groups. Geographical locations of trap haul (latitude and longitude) was used in order to identify clusters around AS Island. Model selection is usually achieved by evaluation of the Bayesian information criterion (BIC) for expectation-maximization (EM) fitted models. Mixture model estimation of the trap location dataset was performed using the ‘‘*mclust*” R library [39,40]. All identified clusters were discretized to simplify the shape and not allow spatial overlapping in inshore-offshore axis, forming zones around AS Island. There are ten model options available in *mclust* R package. Depth and zone attributes were passed to the mark-recapture database for release or recapture depth/zone strata analysis.

To ensure that each point of recapture was assigned to a specific location and avoid duplicity given by the overlap clusterization, the statistical clusters were used as guidance to construct delimited zones projected from coast to offshore.

#### Connectivity around the system

A standard connectivity analysis between zones would imply computing the proportion of individuals recaptured in each zone, given the number tagged in a particular release area at each recapture period. This would yield a large number of statistics, making it hard to interpret, synthesize and decrease sample sizes to very small levels in some zones. We estimated instead 3 parameters (for each recapture period), namely the proportion of individuals recaptured in the same release zone (p1), the proportion of individuals recaptured in contiguous zones (p2), and the proportion of individuals recaptured in zones that were 2 or more zones distant from the release zone (p3). Using a multinomial likelihood function and the number of recaptured individuals for these 3 categories across the release zones we estimated the three parameters for each recapture period. The model was implemented in ADMB software [41].

### Inshore-offshore movement characterization

Distance and movement angle were calculated between the release and recapture sites, with equations taken from Ernst [18].

τi,j=[(Latj−Lati)2+{cos(0.5ABS(Latj+Lati))(Longj−Longi)}2]12 (1)

$$\Gamma_{i,j}=(\theta )arctg\;\left[\{cos\;(0.5ABS({Lat}_{j}-{Lat}_{i}))\times ABS({Long}_{j}-{Long}_{i})\}/ABS({Lat}_{j}-{Lat}_{i})\right]$$ (2)

Where *τ_i,j_* is the distance migrated from latitude/longitude from *i* to *j* and *Γ_i,j_* is the average angle of movement with respect to true north from latitude/longitude *i* to *j*. Possible values for θ are: (a) NE movement (θ = 90-); (b) SE movement (θ = 90+); (c) SW movement (θ = 270-) and (d) NW movement (θ = 270+).

In order to explore shifts between coastal and oceanic regions, centroids were calculated for all the different mark-recapture combinations. Centroids represent the distribution center of recaptured lobsters at the level of geographic aggregation. Comparing such centroids over time, the shifts of the location of population centroids provide a general account of how the population relocated temporally.

To assess the orientation of lobster movement in each zone, a circular statistic approach was used. According to the mark-recapture design, only one tagging process occurs inshore and recapture takes place offshore (M1R2, Table 1), and two tagging processes occur offshore and their recaptures were made inshore (M2R3 and M3R3, Table 1); all other mark-recaptures (M1R1, M1R3, M2R2), in which tagging and recapture occurs in the same depth strata, were not considered in this analysis. For these combinations, movement angles were tested in order to know if the displacement of lobsters occurs inshore or offshore in each zone around the island. Only for these combinations (M1R2, M2R3, M3R3), mean angle vectors were calculated and only distances travelled from 0 to 2.5 km were considered to avoid misinterpretations of lobsters that could move larger distances. These distances were chosen because 2.5 km is the average distance between the lobsters recaptured and the coastline, for those lobsters which were recaptured offshore. The Rayleigh test of uniformity (general unimodal alternative with unknown mean direction and vector length) was used to determine whether orientation deviated significantly from a random distribution for each zone [42]. Circular statistics were conducted in the R platform using *CiscStats* [43] and *circular* [44] packages. The statistical test was deemed significant at p<0.05.

### Inshore-offshore movement modelling

An alternative approach to assess inshore-offshore movement is to estimate the proportion of individuals that moved between depth strata. For this purpose, proportions were calculated between the release depth strata b1, b2 and b3 described before.

These proportions were calculated in two ways; a direct computation based on lobsters moving between depth strata and the implementation of a statistical model to represent movement dynamics and fitted to mark-recapture data. The estimation model attempts to represent the dynamics of tagged lobsters from the first marking period (M1) to the three recapture intervals (R1, R2 and R3).

To reduce the number of model parameters to be estimated in this analysis we grouped the mark recapture data in two larger zones (northern and southern). This divide was established based on differential spatial operational constraints of lobster trap deployment, where in zones 1, 2, 6, 7 and 8 (northern) sea conditions allow fishermen to use the entire extension of the insular platform as opposed to zones 3, 4 and 5, where the predominant wave pattern throughout the fishing season does not allow a regular trap setting in the shallow bathymetric stratum.

Our model incorporated movement, sighting and survival probabilities (S1 File). Survival included natural and fishing mortality, the latter being modeled through observed effort and estimated catchability (*F* = *qE*). Natural mortality (*M*) was assumed 0.18 annual as reported by Arana & Olate [45]. Three catchability parameters (*q*) were estimated according to the three recapture periods (R1, R2 and R3) for each macrozone and fishing effort was assessed as number of traps hauled. Probability of detection was calculated for every release/recapture bathymetry stratum in a matrix depending also on the three recapture periods. Sighting probabilities were modeled as a function of exploitation rate [46,47] and parameters were estimated using a multinomial likelihood function implemented in ADMB [41] software. Estimated movement proportions of tagged lobsters released in different bathymetric strata throughout the three consecutive recapture periods were plotted against observed data.

## Results

### Spatial stratification

The results indicate that there is a time-space dependency in fish trap deployment. The test for proportions rejected the null hypothesis that the number of traps is equally distributed among bathymetric strata (*p*<0.05). Additionally, there are statistical differences in the numbers of traps deployed in the first part of the fishing season versus the second period (Oct-Dec versus Jan-Apr).

**Fig 4:**
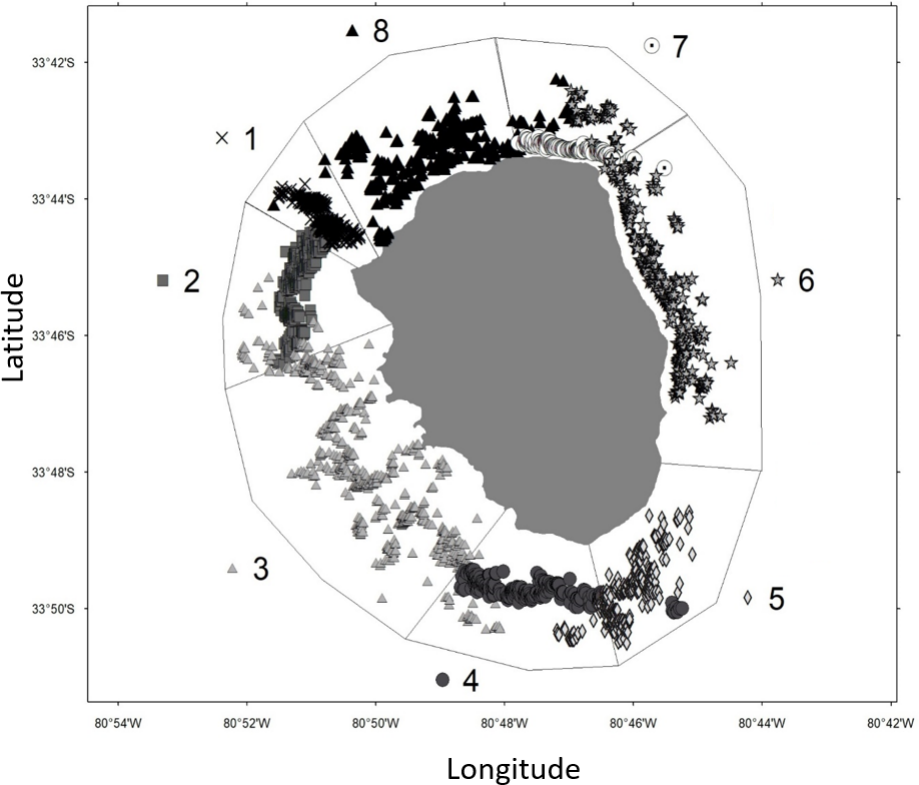
Delimitation of zones from cluster identification around Alejandro Selkirk Island using a multivariate Gaussian mixture model. Number of zones are indicated outside each zone.

The model-based classification method and BIC model selection criteria showed that the best model contains eight clusters (Fig 4), supported by a model in which the estimated covariance matrix is variable in shape, volume and orientation (VVV, 8) This indicates that around AS Island there are 8 conspicuous clusters throughout the fishing season, where traps are deployed. Discretization of these clusters, generates 8 zones around the island, reaching alongshore distances of 0.5 km (zone 1, Fig 4) and larger zones extending from 6 to 8 km in length (zone 3 and 6, Fig 4). Detailed results of other competitive models are shown in S1 Fig and S1 Table.

### Characterization of movement in Alexander Selkirk Island

**Fig 5:**
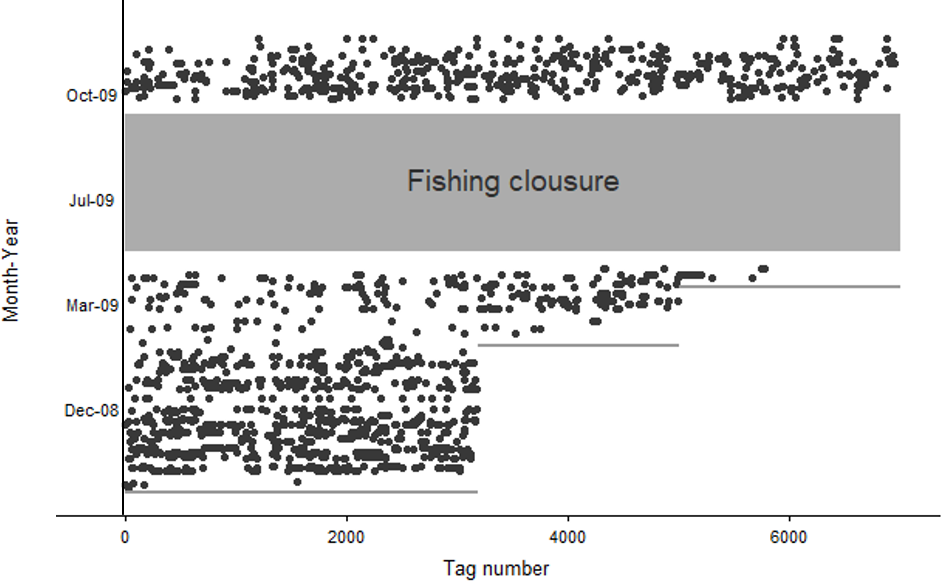
Recapture history by date and tag number. Lines represent mark events and grey rectangle corresponds to fishing closure period from May 15–Sept 30.

Out of 6895 marked lobsters, 1283 were recaptured containing geographic location as part of the biological sampling program throughout a 14-month time period (Fig 5). Recaptures corresponded to 992 males (77%), 237 non-ovigerous females (19%), 53 ovigerous females (4%), and one non-identified individual.

**Fig 6:**
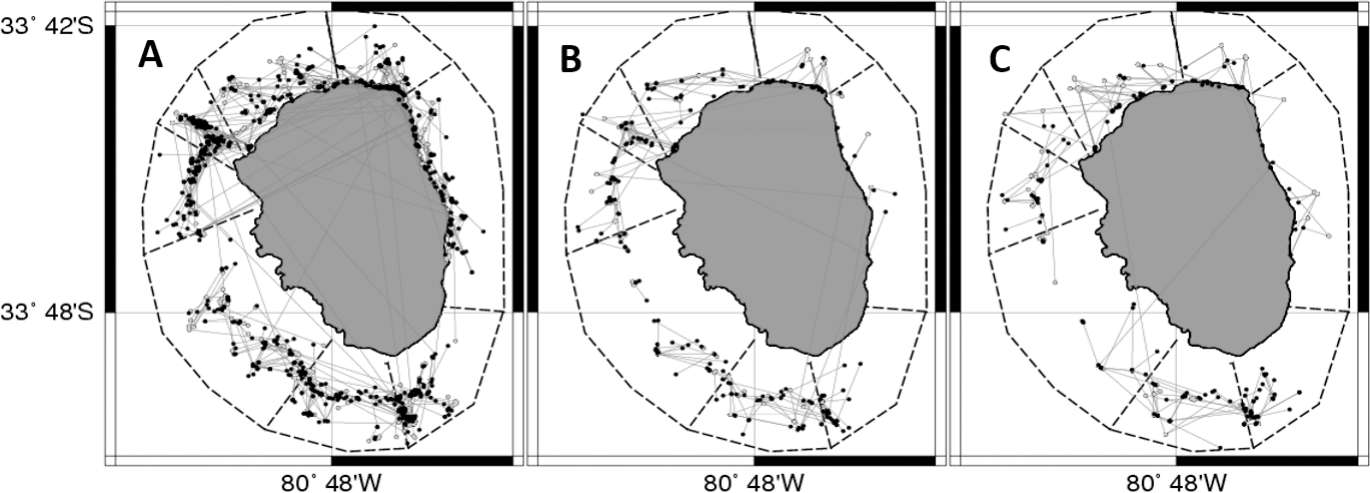
**Euclidean distances recorded from M1(A), M2(B) and M3(C).** Lines correspond to single movements recorded from the three marking events. Distances from all recapture events are shown (M1–912 recaptures, M2–205, and M3–166 recaptures).

From the first marking period (Fig 6A), 912 single recaptures were recorded, the majority of them around zones 1 (n = 145), 6 (n = 144) and 7 (134). From the second marking period (Fig 6B), most (56%) of the 205 recaptured lobsters were caught around the northern and western part of the island, and 44% were caught around zones 3, 4 and 5 located in the southern part. From the third marking period (Fig 6C), 166 lobsters were recaptured in similar areas, mostly around zones 4 (n = 29), 5 (n = 38) and 8 (n = 31), comprising 60% of the total recaptures. Some of the lobsters moved to the opposite side of the island, and therefore Euclidian distances underestimate the travelled distance; nevertheless, this occurred only a few times (32 movements, 2.5% of recaptures).

Throughout the three marking periods, zones 1, 2, 6, 7 and 8 (macrozone 1) and zones 3, 4 and 5 (macrozone 2) showed higher levels of connectivity within each macrozone, highlighted by the discontinuities between zones 2–3 and 5–6 (Fig 6). The comparison of recaptures between those macrozones showed that the proportions of lobsters tagged and recaptured in the same macrozone was always higher than 94%, attributable to some geographic discontinuity detracting connectivity between them.

**Fig 7:**
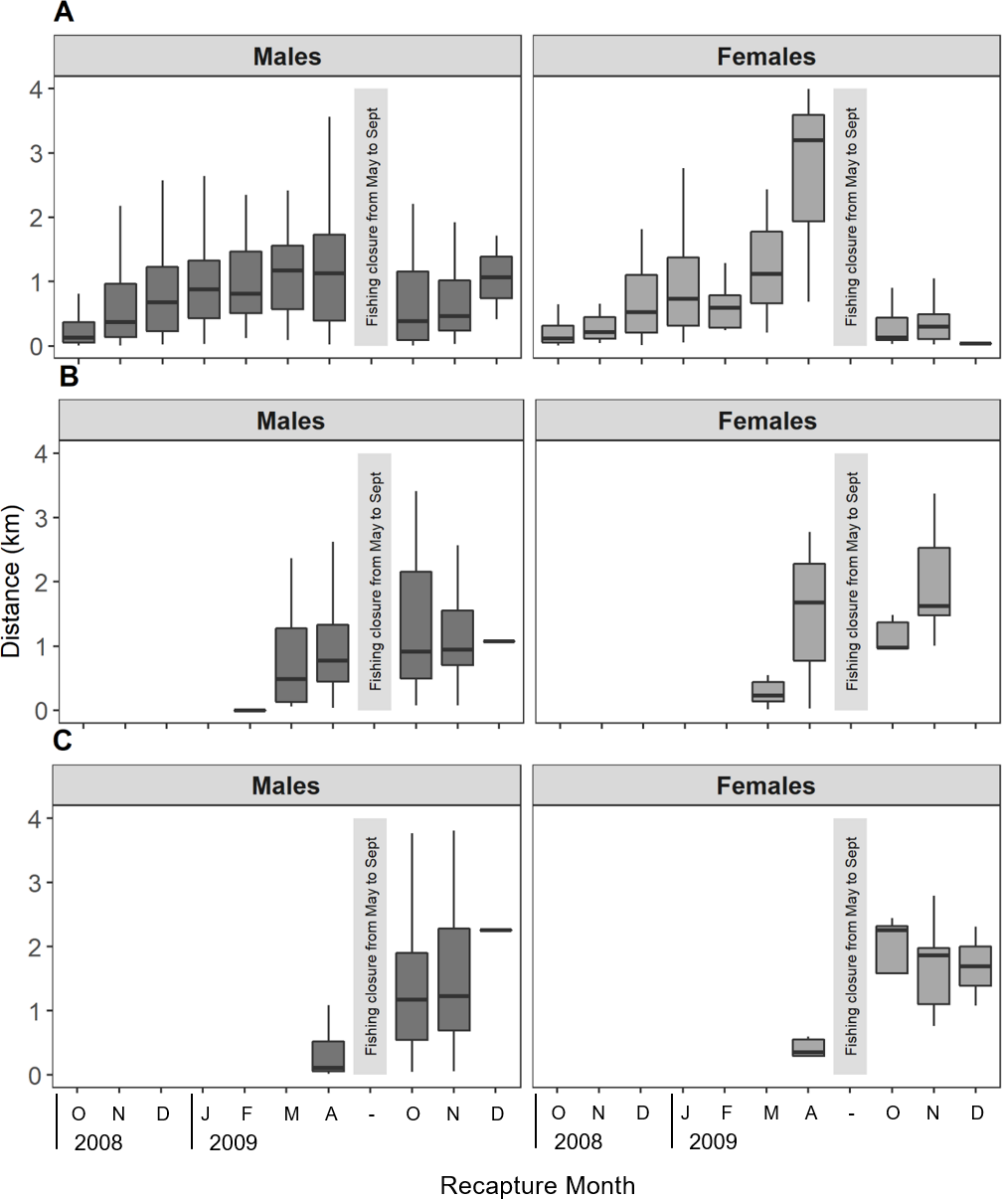
Distance (km) moved by lobsters in AS for different mark-recapture periods. (A) boxes represent lobsters from M1, (B) boxes from M2 and (C) boxes from M3. Distances were calculated for males (left, dark boxes) and females (right, grey boxes).

During the entire study period lobster movements from release to recapture sites (Fig 7) ranged from 0.002 km to 13.03 km ($\bar{x}$ = 1.16 km, sd = 1.70 km). Mean distances travelled by lobsters during the different periods ranged from 0.32 to 2.36 km (Table 2). Distance moved by lobsters from the first tagging event exhibited a remarkable increase throughout the 2008/09 fishing season, reaching maximum levels in March-April of 2009 for both sexes (Fig 7A). After the winter fishing closure, in October 2009, the distances to the mark site again became shorter, showing a similar pattern to October 2008. In February and April tagging events (Fig 7B and 7C), in which lobsters were tagged mainly offshore, distances recorded by males between mark and recapture sites experienced an increase after the winter fishing closure. For female lobsters this pattern is not clear, likely because of the lower sample size.

**Table 2:** Mean distance and standard deviation in kilometers for marking and recapture periods in males, females and ovigerous females.

	Males	Females	Ovigerous
M1			
R1	0.90 (1.68)	0.51 (0.86)	0.32 (0.44)
R2	1.59 (1.90)	1.22 (1.66)	2.19 (2.76)
R3	1.03 (1.55)	0.85 (2.18)	1.13 (1.57)
M2			
R2	0.83 (1.10)	1.05 (0.66)	0.70 (0.82)
R3	2.03 (2.18)	2.36 (2.35)	-
M3			
R3	1.66 (1.02)	1.90 (1.54)	-

Mean distances travelled by males and females present no statistical differences by marking event (*t* = -3.14, *p*>0.05). In the case of ovigerous females, two of them were recaptured in R3 arising from marking periods M2 and M3 (0.49 and 9.83 km respectively), nevertheless, this could not be statistically compared to non-ovigerous females due to the small number of recaptured ovigerous females.

Movement distances recorded from M1 to R1 and from M1 to R2 were statistically different for sex-combined data (*t* = -5.09; *p*<0.001). Also, there were statistical differences between recapture periods 2 and 3 of lobsters from the first tagging period (*t* = 2.90; *p* = 0.004). Finally, comparing distances moved from M1 to recapture periods 1 and 3, there are no statistical differences (*t* = -0.92; *p* = 0.360). From M2 to recapture period 2 and 3, there were statistical differences in distance moved (*t* = -5.01; *p*<0.001) and also differences were found between tagging period 3 and the recapture periods occurring before and after the fishing closure (*t* = -6.97; *p*<0.001)

### Connectivity around Alexander Selkirk Island

Movements for every mark-recapture period showed a similar pattern, with higher proportions of recapture within the release zones. Secondly, proportions of recapture occurring in contiguous zone were between 12% and 30%, and the lowest proportion of recapture were found in remote zones (0% to 10%; Table 3).

**Table 3:** Proportions of recapture and standard error from mark-recapture events. p1 –proportions recaptured within release zones, p2 –proportions recaptured in contiguous zone to release zone, and p3 –proportions recaptured in remote zones.

Mark-recapture event	Proportions	Point estimate	Standard error
M1—R1	p1	0.81	0.02
	p2	0.16	0.02
	p3	0.02	0.01
M1—R2	p1	0.77	0.03
	p2	0.17	0.02
	p3	0.06	0.01
M1—R3	p1	0.84	0.03
	p2	0.12	0.03
	p3	0.05	0.02
M2—R2	p1	0.83	0.03
	p2	0.17	0.03
	p3	0.00	0.00
M2—R3	p1	0.60	0.05
	p2	0.30	0.05
	p3	0.10	0.03
M3—R3	p1	0.72	0.04
	p2	0.24	0.04
	p3	0.04	0.02

During the M1R1, M1R3 and M2R2 periods, the proportions of recapture in the same zones were higher than 80%. For periods in which marking took place offshore (M2R3, M3R3) higher proportions of recaptures were found in contiguous zones, ranging between 24 and 30% (Table 3).

### Inshore-offshore movement characterization

**Fig 8:**
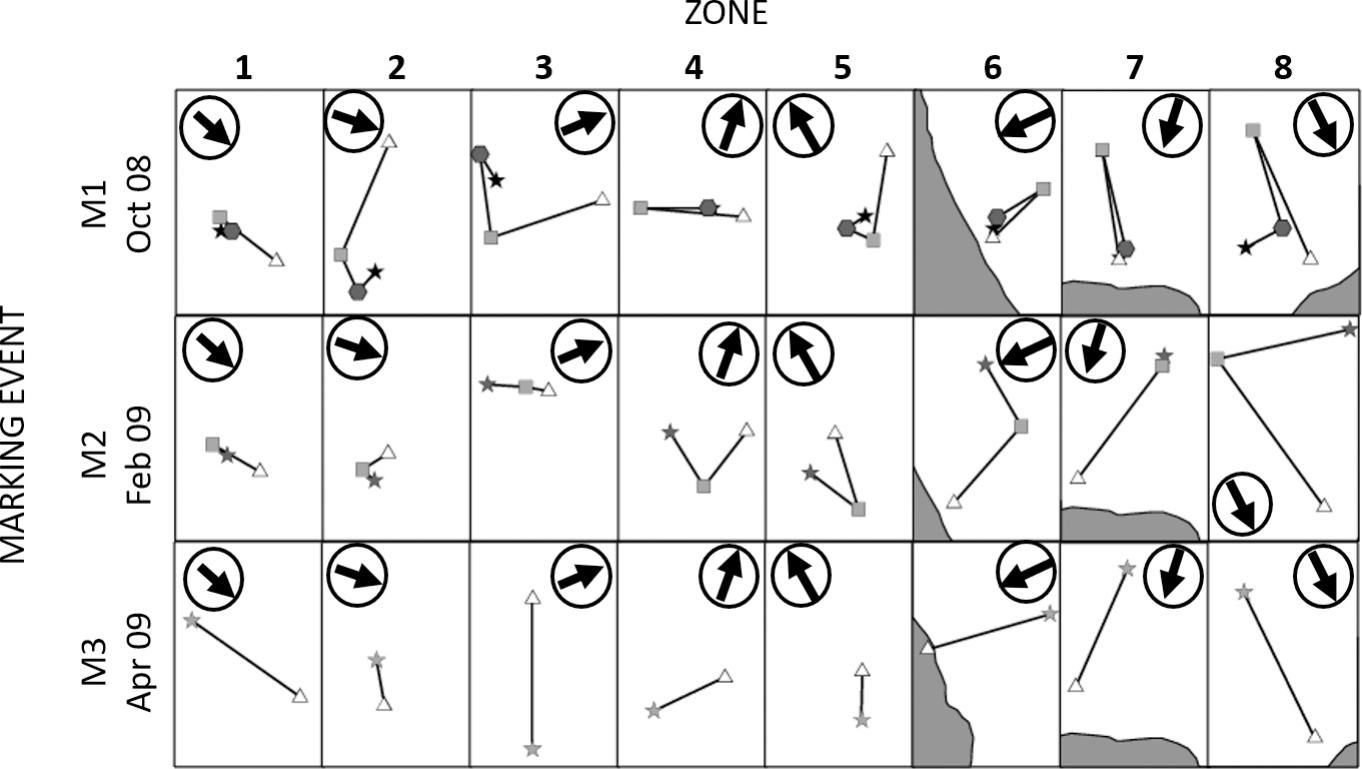
Centroid shifts for three marking periods and their successive recapture periods. Stars (★) represents tagging M1, M2 and M3 (rows), hexagons (#) are the first recapture period R1 (Oct-Dec 2008), squares (■) the second recapture period R2 (Jan-Apr 2009), and triangles (▲) the third recapture period R3 (Oct-Nov 2009). Black circled arrows indicate direction to the coast.

Centroids of lobsters in each zone varied between mark and recapture periods. For the first tagging event (first row, Fig 8), lobsters recaptured during R1 appear to be near the release area (hexagon) in each zone. Besides, lobsters recaptured during R2 (squares) were less close to the release point (star) and lobsters recaptured during R3 (triangles) tended to be close to the coast in almost all zones. This pattern indicates that movement after the first tagging event were initially away from the coastline, but after that the lobsters returned inshore. Some offshore shifts in the spatial distribution of R1 and R2 centroids are evident for some areas, but most conspicuously for zones 6, 7 and 8. For the second mark event (second row, Fig 8), lobsters recaptured during R2 were closer to the release centroid than recaptures from R3, showing a pronounced inshore displacement in almost all zones, as occurred also for the lobsters tagged during the third period (third row, Fig 8). Additional explorations on the displacement angle between mark-recapture periods are presented in S2 Fig.

In mark recapture period M1R2 all the mean angles were oriented offshore (Table 4); there were statistical differences that failed to reject the null hypothesis of uniformity in favor of directionality, especially in zones 5, 6, 7 and 8 (south, east and north of the island *p*<0.05). Furthermore, for the mark-recapture periods M2R3 and M3R3, the mean angles of all the zones were strongly directed to inshore waters and the same test rejected the null hypothesis of uniformity especially in zones 7 and 8 (*p*<0.05; Table 4).

**Table 4:** Characterization of movement angles comprising inshore-offshore sites of mark and recapture, and Rayleigh test estimations by zone.

Period	Zones	Sample size	Mean angle	Windrose angle	R-test uniformity
	1	57	315.0	NW	0.892
	2	17	242.2	SW	0.087
	3	32	206.1	SW	0.059
M1–R2	4	20	232.3	SW	0.182
In-Off	5	33	188.8	S	0.018
	6	24	62.2	NE	0.007
	7	22	356.9	N	0.000
	8	18	315.4	NW	0.005
	1	15	126.1	SE	0.046
	2	4	42.5	NE	0.421
	3	5	83.0	E	0.045
M2–R3	4	12	65.8	NE	0.011
Off-In	5	7	341.0	N	0.469
	6	2	208.7	SW	0.137
	7	11	196.7	S	0.000
	8	10	121.0	SE	0.037
	1	1	175.7	S	0.512
	2	16	46.1	NE	0.124
	3	10	70.3	E	0.823
M3–R3	4	18	43.2	NE	0.023
Off-In	5	27	348.0	N	0.000
	6	10	253.7	W	0.001
	7	11	194.2	S	0.002
	8	21	130.4	SE	0.000

### Inshore-offshore movement modelling

**Fig 9:**
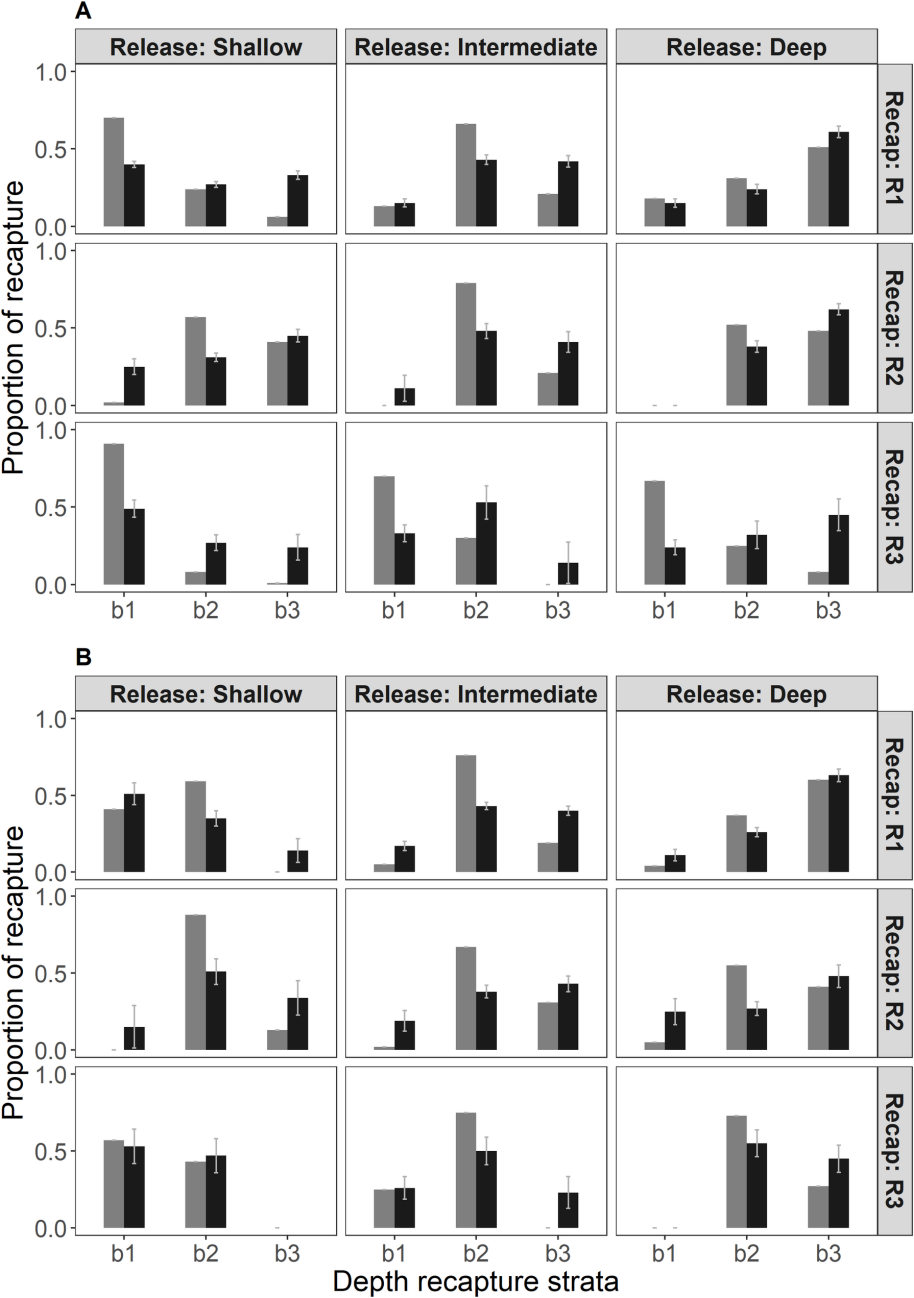
**Estimated (black bars) and observed (grey bars) movement proportions for recaptures in northern (upper panel, A) and southern (lower panel, B) macrozones**. b1 –shallow (0–50 meters), b2 –intermediate (51–100 meters), and deep level (>101 meters).

In the northern macrozone (Fig 9A) lobsters released in shallow waters were observed in higher proportions in shallow (b1) and intermediate (b2) strata during the first recapture period, higher proportions in intermediate and deep (b3) strata during the second recapture interval and highest again in shallow stratum during the third recapture period. This pattern was more conspicuous for observed than model predicted proportions. Lobsters released in the intermediate stratum remained at higher proportions in this zone for the first and second recapture periods, but were observed at higher levels in shallow areas during the last recapture term. There was a marked difference between observed and model quantities for the last recapture interval. Finally, lobsters released in deep waters remained mostly in intermediate and deep strata during the first and second recapture periods and displayed a conspicuous increase in shallow waters during the last recapture period. This pattern is not captured by the model, which predicts lower proportions in shallow waters during the third recapture period.

The southern macrozone (Fig 9B) showed in general a similar pattern, but with some differences. Lobsters released in shallow areas were found in high proportions in the intermediate stratum during the three recapture periods and lobsters released in the three strata were observed at smaller proportions in shallow waters during the third recapture period.

Main differences between model estimates and data proportions of recapture can be attributed to model attempts to deal with lower detection rates given by lower fishing effort. The model takes into account the differences in fishing effort between depth strata and penalizes strata with less coverage bringing them higher probabilities of recapture and strata with higher fishing effort with less recapture probabilities.

## Discussion

### Spatial stratification

The eight clusters determined by the model of the AS system are well delimited and some of them coincide with seascape features identified by fishermen [35]. This stratification helped to analyze movements of spiny lobsters around the island with a new approach never used before in this system, providing a better delimitation than statistical zones used in previous analyses [35]. We used spatial location of individual discrete fishing spots employed regularly by fishermen [35] and the zones delimited after the cluster analyses as a proxy of resource distribution. These clusters vary in size reaching extensions from 0.5 to 6–8 km, generally with an alongshore orientation.

Many factors could influence the spatial allocation of fishing effort, such as (a) spatial distribution of the fishery stocks, (b) the differential value of various target species, (c) sea and weather conditions, (d) social factors such as local traditions or agreements among stakeholders and managers, and/or, (e) the location of a MPA with respect to fishing ports [48, 49]. In nature, organisms are distributed neither uniformly nor at random. Rather they are aggregated in patches or other kinds of spatial arrangements [50]. Animals targeted by any fisheries form associations depending on the species [51], size and age class [52], seasonality [53] or habitat association [54]. As the occurrence of the targeted species is often reflected in the spatial patterns of the corresponding fishing activity [55], fishing effort data can be characterised by a high level of spatial heterogeneity. Considering fishing effort points for the analysis, the method applied in this study, gives better estimates of the number of clusters, lower classification error rates, more parsimonious clustering models, and hence easier interpretation and visualization than clustering using all of the available variables (temporal, biological, operational, fishing variables) [56,57].

### Seasonal movements in AS system

Numerous studies have reported long-distance nomadic movements of lobsters, particularly in *Jasus edwardsii* [58–63], *J*. *lalandii* [64–66] and *J*. *verreauxi* [67,68]. Nomadic movements are dispersal events often associated with changes in habitat during ontogenetic development or induced by high densities of lobsters relative to local resources [19]. These movements could be less marked around oceanic islands because of their smaller size and their shorter insular shelves, and in areas with abundant food and shelter [69]. Also, it has been speculated that inshore–offshore movements may be environmentally driven, with lobsters following specific temperature regimes that are optimal for rapid growth and embryo development [70].

**Fig 10:**
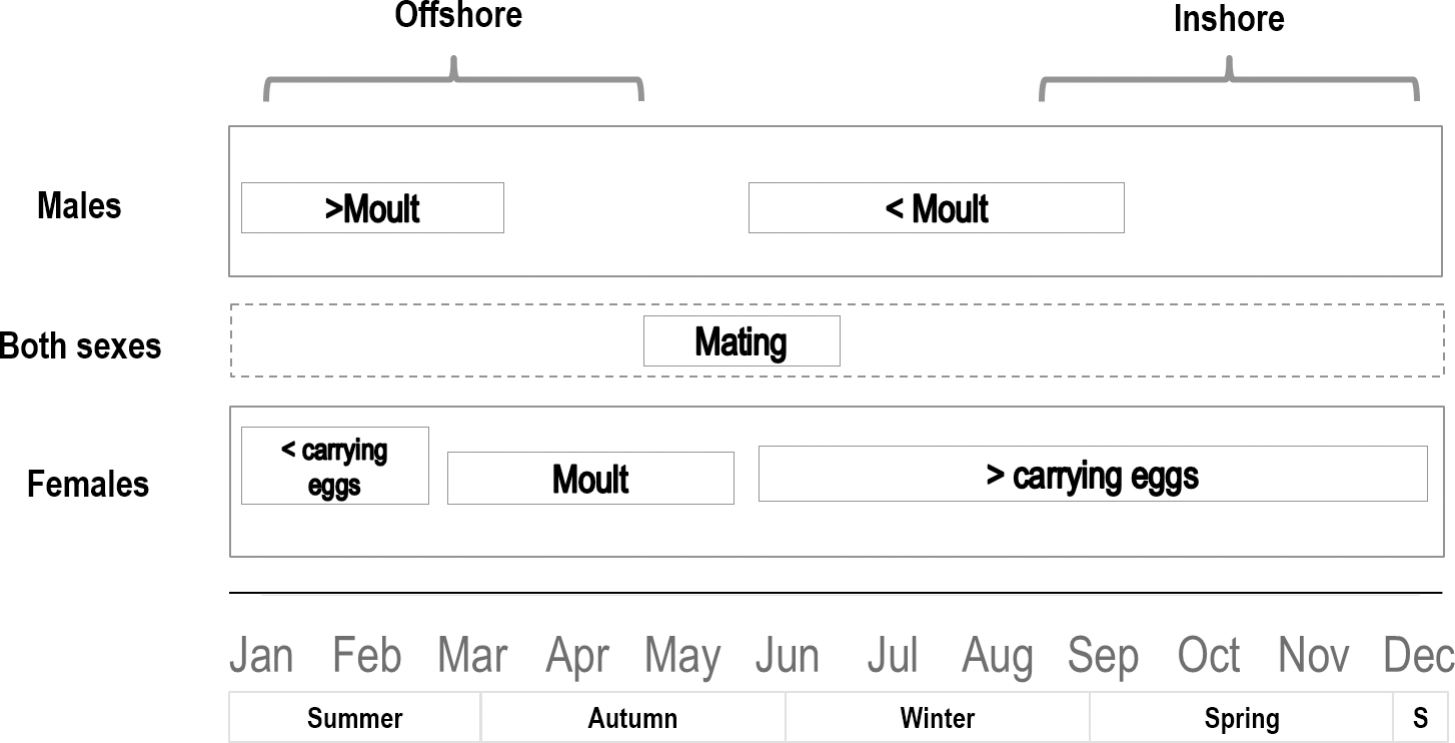
Diagram of inshore-offshore processes occurring at temporal and seasonal scales for males, females and combined sex.

The spatial pattern of recoveries offers some enlightenment. During this study, tag recaptures were predominantly in shallow waters after the fishing closure and in deeper waters during the austral summer-autumn period, showing a remarkable seasonality. In view of these findings, movements of *J*. *frontalis* in AS Island could be associated with different biological processes as detailed in Fig 10. Recorded movements of tagged lobsters in this study were mostly between 1 to 2 km which is similar to distances moved reported for *J*. *edwardsii* [61,63,71] and *Panulirus interruptus* that live on narrow coastal shelves, and contrast with longer distances reported for *P*. *argus* and *P*. *cygnus* [10].

According to biological processes, female ecdysis occurs in mid-April to June shortly before the mating period, which takes place between May and June [72,73]. Male ecdysis occurs twice a year in summer and winter [37]. These patterns are closely related to *J*. *edwardsii*, where males undergo ecdysis in spring between October and November, while female ecdysis occurs in autumn (between April and June) and mating occurs afterwards [74]. After mating, *J*. *frontalis* females carry eggs between July and February with a peak observed in October-December in the Robinson Crusoe-Santa Clara system [72,73], when lobsters are fully exploited but safeguarding the harvesting of egg-bearing females.

Movement activity of female *J*. *edwardsii* is low during the mating season (April) but increases over the brooding season (winter) to peak around the time of larval release [74]. The increase in activity during the brooding season was due to the movement of females into deeper offshore areas. Having found that female *J*. *edwardsii* aggregate around the edge of deep reefs toward the end of the egg-bearing season, McKoy & Leachman [75] proposed that aggregation in areas with strong tidal currents may facilitate the rapid dispersal of newly hatched larvae away from reef-dwelling planktivorous predators. This pattern could not be affirmed in this study because ovigerous females of *J*. *frontalis* moved very little. Nevertheless, the low quantity of ovigerous females tagged inshore in October (M1) and offshore in February and April (M2 and M3) and consequently the low rates of recapture, could be explained by the low probability of ovigerous females to move and enter the traps during embryo incubation, specially females carrying late state eggs [76]. Furthermore, a major proportion of larger females carrying eggs in the AS system could occur earlier in this system (Aug-Sept), i.e. before the fishing season, as reported by Ernst [77,78]. The significance of these migrations (horizontal and vertical) in reproduction is not yet clear for this system, although the movement of egg-bearing females towards areas of high water movement presumably facilitates larval dispersal [11,15,75].

### Factors driving movements of *Jasus frontalis*

The biological processes underpinning seasonal inshore-offshore movement patterns of *J*. *frontalis* appear to be closely linked to reproductive and moulting behaviour as occurs in congeneric species and even in the whole palinurid family. In *J*. *lalandii*, the movements and their interannual variation were directly related to the inshore presence of water with very low levels of dissolved oxygen [64]. Inshore-offshore migrations made by *Homarus americanus* in the Gulf of Maine have also been observed in some regional areas, apparently in response to strong winds and associated turbulence [79], with temporal differences in movement patterns between sexes [80]. In *Panulirus argus*, autumnal storms were associated with mass migrations [81,82]. These storms have a profound effect on the shallow (1–10 m) waters of the Great Bahama Bank surrounding Bimini (Bahamas) where the majority of the migratory population originates.

Temperature is one of the environmental factors that has major effects on growth and survival in crustaceans [83]. In lobsters, water temperature also affects behaviour [84], and their motivation to find food. In *J*. *edwardsii*, it has been documented that temperature is determinant in catchability and also over biological processes such as moult and mating among others [85]. Ernst et al. [35] reported high variations in near bottom temperature between 14°C and 19°C throughout the same fishing season analyzed in this study (2008/09), similar to temperatures reported by Ziegler et al. [85], which allows inferring that temperature also plays a key role on biological patterns and ontogenetic migrations of *J*. *frontalis*.

### Implications for fisheries activities and management

For the Juan Fernández fishery previous contributions have demonstrated the importance and effectiveness of regulating access and spatial effort by informal but tightly structured local practices [21,22,35]. The informal sea tenure system (*“Marcas”*) tie fishermen to a collection of individually owned fishing spots, where they exert exclusive access to the lobster resource. The results of this study show high retention in the identified clusters and an important seasonal inshore-offshore movement throughout the fishing season. Most fishermen have *Marcas* in inshore and offshore locations, exploiting lobsters across their full bathymetric range for particular zones around the island.

The results of this study indicate that lobsters are probably transgressing fishing territories of individual fishermen through seasonal inshore/offshore and between cluster movements, with the former being more relevant. Research should be oriented to clarify if the individual discrete fishing spots for particular fishermen have inshore and offshore representation, as a strategy to cover the full bathymetric range of moving lobsters.

### Conclusions and outlook

Movement patterns have been described for most *Jasus* species around the southern hemisphere [10,25]. These movements have been described mostly as a seasonal migration from inshore to offshore areas. Those migrations occur in subtropical and temperate areas and are generally attributed to environmental stimuli interacting with internal physiological events [10]. This study focused on *J*. *frontalis* from AS Island, providing solid evidence on population clustering, alongshore connectivity, and inshore-offshore movement based on a high frequency sampling of mark-recapture data and a conceptual model to establish comparisons with congeneric species. Lobsters showed high degree of clustering with 8 clusters (zones) of less than 8 km in length, about 60–80% of retention in those patches and a marked seasonal inshore-offshore movement.

Passive tags and large-scale opportunistic sampling (during fishing trips) have provided a great opportunity to complete an island-wide lobster movement study. Causality and finer-scale timing of movement may be conspicuously improved by using acoustic and archival tags. This allows the continuous collection of environmental and depth data to obtain a better linkage between reproduction and inshore-offshore seasonal movement.

## Supporting information

S1 Table Best models selected by cluster classification (mclust) and comparison of BIC values. (DOCX)

S1 File Equations of mark-recapture model. Equations are identical for north and south macrozones.
(DOCX)

S1 Fig BIC from mclust for the ten available model parametrizations and up to 9 clusters for the dataset. Different symbols and line types encode different model parametrizations. The best model is taken to be the one with the highest BIC among the fitted models (black arrow).
(DOCX)

S2 Fig **Angles direction for each zone around AS Island in mark recapture periods A (M1R2), B (M2-R3) and C (M3-R3)**. Line around the circle represents the underlying circular distributions of displacement angles.
(DOCX)
