# Supplementary material for: Spatio-temporal migratory dynamics of Jasus frontalis (Milne Edwards, 1837) in Alexander Selkirk Island, Juan Fernández archipelago, Chile
Source: PLoS One. 2018 Jul 25;13(7):e0200146. doi: 10.1371/journal.pone.0200146 (PMC6059422; doi:10.1371/journal.pone.0200146)
Supplement: S1 Table — (DOCX) [file pone.0200146.s001.docx]

1. **Best models selected by cluster classification (mclust) and comparison of BIC values.**

|  | VVV,8 | VVV,9 | VEV,9 |
| --- | --- | --- | --- |
| BIC | 33526.13 | 33506.75 | 33217.03 |
| BIC diff | 0 | -19.38 | -309.10 |
| *Values for three of the eight cluster models | | | |
