# Supplementary material for: Spatio-temporal migratory dynamics of Jasus frontalis (Milne Edwards, 1837) in Alexander Selkirk Island, Juan Fernández archipelago, Chile
Source: PLoS One. 2018 Jul 25;13(7):e0200146. doi: 10.1371/journal.pone.0200146 (PMC6059422; doi:10.1371/journal.pone.0200146)
Supplement: S1 File — Equations are identical for north and south macrozones. (DOCX) [file pone.0200146.s002.docx]

**Mark/recapture model equations.**

$\Omega_{i,j}^{k}$=probability of detection released in *i*, recaptured in *j*, in period *k*

$k$= $\left\{ 1,2,3 \right\}$ ; 1: Oct-08 to Dec-08; 2: Jan-09 to Apr09; 3: Oct-09 to Nov-09

$i,j$= $\left\{ 1,2,3 \right\}$ ; 1: 0-50m; 2: 51-100m; 3: >100m

$m_{i,j}$=Probability of finding in *j* from *i*

$f_{i}^{k}$=total effort in site I in period k

$M$= $\left\{ 1,2,3,4 \right\}$ natural mortality. (Annual 0.18, monthly 0.015, 1: Oct08-Dec08 0.045, 2: Jan09-15thMay09 0.0675, 3: Oct09-Nov09 0.03, 4: winter fishing closure 0.0675)

$q^{k}$=catchability

$F_{i}^{k}=q^{k}*f_{i}^{k}$= Fishing mortality

Tag group 1 🡪 Marked on October 2008

1. Recaptured between Oct 08 to Dec 09 – Macrozone North/South

Lobsters marked in depth strata 1 during October 2008 in north macrozone, recovered in sites 1 (coastal), 2 (middle) and 3 (deeper). On each equation, natural mortality applied was 0.045

$\Omega_{1,1}^{1}$ = $m_{1,1}^{1}*\frac{F_{1}^{1}}{F_{1}^{1}+M_{1}}*(1-{exp}^{\left( -\left( F_{1}^{1}+M_{1} \right) \right)})$

$\Omega_{1,2}^{1}$ = $m_{1,2}^{1}*\frac{F_{2}^{1}}{F_{2}^{1}+M_{1}}*(1-{exp}^{\left( -\left( F_{2}^{1}+M_{1} \right) \right)})$

$\Omega_{1,3}^{1}$ = $m_{1,3}^{1}*\frac{F_{3}^{1}}{F_{3}^{1}+M_{1}}*(1-{exp}^{\left( -\left( F_{3}^{1}+M_{1} \right) \right)})$

Lobsters marked in depth strata 2 during October 2008 in north macrozone, recovered in sites 1 (coastal), 2 (middle) and 3 (deeper). On each equation, natural mortality applied was 0.045

$\Omega_{2,1}^{1}$ = $m_{2,1}^{1}*\frac{F_{1}^{1}}{F_{1}^{1}+M_{1}}*(1-{exp}^{\left( -\left( F_{1}^{1}+M_{1} \right) \right)})$

$\Omega_{2,2}^{1}$ = $m_{2,2}^{1}*\frac{F_{2}^{1}}{F_{2}^{1}+M_{1}}*(1-{exp}^{\left( -\left( F_{2}^{1}+M_{1} \right) \right)})$

$\Omega_{2,3}^{1}$ = $m_{2,3}^{1}*\frac{F_{3}^{1}}{F_{3}^{1}+M_{1}}*(1-{exp}^{\left( -\left( F_{3}^{1}+M_{1} \right) \right)})$

Lobsters marked in depth strata 3 during October 2008 in north macrozone, recovered in sites 1 (coastal), 2 (middle) and 3 (deeper). On each equation, natural mortality applied was 0.045

$\Omega_{3,1}^{1}$ = $m_{3,1}^{1}*\frac{F_{1}^{1}}{F_{1}^{1}+M_{1}}*(1-{exp}^{\left( -\left( F_{1}^{1}+M_{1} \right) \right)})$

$\Omega_{3,2}^{1}$ = $m_{3,2}^{1}*\frac{F_{2}^{1}}{F_{2}^{1}+M_{1}}*(1-{exp}^{\left( -\left( F_{2}^{1}+M_{1} \right) \right)})$

$\Omega_{3,3}^{1}$ = $m_{3,3}^{1}*\frac{F_{3}^{1}}{F_{3}^{1}+M_{1}}*(1-{exp}^{\left( -\left( F_{3}^{1}+M_{1} \right) \right)})$

1. Recaptured between Jan 09 to Apr 09 – Macrozone North/South

Lobsters marked in depth strata 1 during October 2008

$\Omega_{1,1}^{2}$ = $m_{1,1}^{2}*\frac{F_{1}^{2}}{F_{1}^{2}+M_{2}}*\left( 1-{exp}^{\left( -\left( F_{1}^{2}+M_{2} \right) \right)} \right)*({exp}^{\left( -\left( F_{i}^{1}+M_{1} \right) \right)})$

$\Omega_{1,2}^{2}$ = $m_{1,2}^{2}*\frac{F_{2}^{2}}{F_{2}^{2}+M_{2}}*\left( 1-{exp}^{\left( -\left( F_{2}^{2}+M_{2} \right) \right)} \right)*({exp}^{\left( -\left( F_{i}^{1}+M_{1} \right) \right)})$

$\Omega_{1,3}^{2}$ = $m_{1,3}^{2}*\frac{F_{3}^{2}}{F_{3}^{2}+M_{2}}*\left( 1-{exp}^{\left( -\left( F_{3}^{2}+M_{2} \right) \right)} \right)*({exp}^{\left( -\left( F_{i}^{1}+M_{1} \right) \right)})$

Lobsters marked in depth strata 2 during October 2008

$\Omega_{2,1}^{2}$ = $m_{2,1}^{2}*\frac{F_{1}^{2}}{F_{1}^{2}+M_{2}}*\left( 1-{exp}^{\left( -\left( F_{1}^{2}+M_{2} \right) \right)} \right)*({exp}^{\left( -\left( F_{i}^{1}+M_{1} \right) \right)})$

$\Omega_{2,2}^{2}$ = $m_{2,2}^{2}*\frac{F_{2}^{2}}{F_{2}^{2}+M_{2}}*\left( 1-{exp}^{\left( -\left( F_{2}^{2}+M_{2} \right) \right)} \right)*({exp}^{\left( -\left( F_{i}^{1}+M_{1} \right) \right)})$

$\Omega_{2,3}^{2}$ = $m_{2,3}^{2}*\frac{F_{3}^{2}}{F_{3}^{2}+M_{2}}*\left( 1-{exp}^{\left( -\left( F_{3}^{2}+M_{2} \right) \right)} \right)*({exp}^{\left( -\left( F_{i}^{1}+M_{1} \right) \right)})$

Lobsters marked in depth strata 3 during October 2008

$\Omega_{3,1}^{2}$ = $m_{3,1}^{2}*\frac{F_{1}^{2}}{F_{1}^{2}+M_{2}}*\left( 1-{exp}^{\left( -\left( F_{1}^{2}+M_{2} \right) \right)} \right)*({exp}^{\left( -\left( F_{i}^{1}+M_{1} \right) \right)})$

$\Omega_{3,2}^{2}$ = $m_{3,2}^{2}*\frac{F_{2}^{2}}{F_{2}^{2}+M_{2}}*\left( 1-{exp}^{\left( -\left( F_{2}^{2}+M_{2} \right) \right)} \right)*({exp}^{\left( -\left( F_{i}^{1}+M_{1} \right) \right)})$

$\Omega_{3,3}^{2}$ = $m_{3,3}^{2}*\frac{F_{3}^{2}}{F_{3}^{2}+M_{2}}*\left( 1-{exp}^{\left( -\left( F_{3}^{2}+M_{2} \right) \right)} \right)*({exp}^{\left( -\left( F_{i}^{1}+M_{1} \right) \right)})$

1. Recaptured between Oct 09 to Nov 09 – Macrozone North/South

Lobsters marked in depth strata 1 during October 2008

$\Omega_{1,1}^{3}$ = $m_{1,1}^{3}*\frac{F_{1}^{3}}{F_{1}^{3}+M_{3}}*\left( 1-{exp}^{\left( -\left( F_{1}^{3}+M_{3} \right) \right)} \right)*({exp}^{\left( -\left( F_{i}^{1}+M_{1} \right) \right)})*({exp}^{\left( -\left( F_{i}^{2}+M_{2} \right) \right)})*({exp}^{\left( -M_{4} \right)})$

$\Omega_{1,2}^{3}$ = $m_{1,2}^{3}*\frac{F_{2}^{3}}{F_{2}^{3}+M_{3}}*\left( 1-{exp}^{\left( -\left( F_{2}^{3}+M_{3} \right) \right)} \right)*({exp}^{\left( -\left( F_{i}^{1}+M_{1} \right) \right)})*({exp}^{\left( -\left( F_{i}^{2}+M_{2} \right) \right)})*({exp}^{\left( -M_{4} \right)})$

$\Omega_{1,3}^{3}$ = $m_{1,3}^{3}*\frac{F_{3}^{3}}{F_{3}^{3}+M_{3}}*\left( 1-{exp}^{\left( -\left( F_{3}^{3}+M_{3} \right) \right)} \right)*({exp}^{\left( -\left( F_{i}^{1}+M_{1} \right) \right)})*({exp}^{\left( -\left( F_{i}^{2}+M_{2} \right) \right)})*({exp}^{\left( -M_{4} \right)})$

Lobsters marked in depth strata 2 during October 2008

$\Omega_{2,1}^{3}$ = $m_{2,1}^{3}*\frac{F_{1}^{3}}{F_{1}^{3}+M_{3}}*\left( 1-{exp}^{\left( -\left( F_{1}^{3}+M_{3} \right) \right)} \right)*({exp}^{\left( -\left( F_{i}^{1}+M_{1} \right) \right)})*({exp}^{\left( -\left( F_{i}^{2}+M_{2} \right) \right)})*({exp}^{\left( -M_{4} \right)})$

$\Omega_{2,2}^{3}$ = $m_{2,2}^{3}*\frac{F_{2}^{3}}{F_{2}^{3}+M_{3}}*\left( 1-{exp}^{\left( -\left( F_{2}^{3}+M_{3} \right) \right)} \right)*({exp}^{\left( -\left( F_{i}^{1}+M_{1} \right) \right)})*({exp}^{\left( -\left( F_{i}^{2}+M_{2} \right) \right)})*({exp}^{\left( -M_{4} \right)})$

$\Omega_{2,3}^{3}$ = $m_{2,3}^{3}*\frac{F_{3}^{3}}{F_{3}^{3}+M_{3}}*\left( 1-{exp}^{\left( -\left( F_{3}^{3}+M_{3} \right) \right)} \right)*({exp}^{\left( -\left( F_{i}^{1}+M_{1} \right) \right)})*({exp}^{\left( -\left( F_{i}^{2}+M_{2} \right) \right)})*({exp}^{\left( -M_{4} \right)})$

Lobsters marked in depth strata 3 during October 2008

$\Omega_{3,1}^{3}$ = $m_{3,1}^{3}*\frac{F_{1}^{3}}{F_{1}^{3}+M_{3}}*\left( 1-{exp}^{\left( -\left( F_{1}^{3}+M_{3} \right) \right)} \right)*({exp}^{\left( -\left( F_{i}^{1}+M_{1} \right) \right)})*({exp}^{\left( -\left( F_{i}^{2}+M_{2} \right) \right)})*({exp}^{\left( -M_{4} \right)})$

$\Omega_{3,2}^{3}$ = $m_{3,2}^{3}*\frac{F_{2}^{3}}{F_{2}^{3}+M_{3}}*\left( 1-{exp}^{\left( -\left( F_{2}^{3}+M_{3} \right) \right)} \right)*({exp}^{\left( -\left( F_{i}^{1}+M_{1} \right) \right)})*({exp}^{\left( -\left( F_{i}^{2}+M_{2} \right) \right)})*({exp}^{\left( -M_{4} \right)})$

$\Omega_{3,3}^{3}$ = $m_{3,3}^{3}*\frac{F_{3}^{3}}{F_{3}^{3}+M_{3}}*\left( 1-{exp}^{\left( -\left( F_{3}^{3}+M_{3} \right) \right)} \right)*({exp}^{\left( -\left( F_{i}^{1}+M_{1} \right) \right)})*({exp}^{\left( -\left( F_{i}^{2}+M_{2} \right) \right)})*({exp}^{\left( -M_{4} \right)})$
