# Supplementary material for: Spatio-temporal migratory dynamics of Jasus frontalis (Milne Edwards, 1837) in Alexander Selkirk Island, Juan Fernández archipelago, Chile
Source: PLoS One. 2018 Jul 25;13(7):e0200146. doi: 10.1371/journal.pone.0200146 (PMC6059422; doi:10.1371/journal.pone.0200146)
Supplement: S1 Fig — Different symbols and line types encode different model parametrizations. The best model is taken to be the one with the highest BIC among the fitted models (black arrow). (DOCX) [file pone.0200146.s003.docx]

**Supporting information 2**


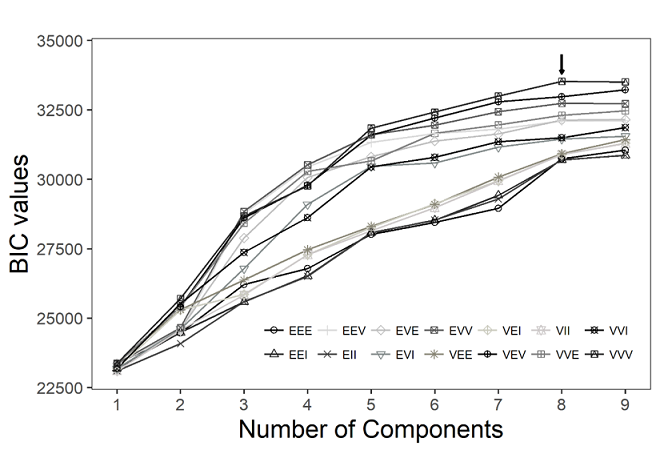


1. BIC from mclust for the ten available model parametrizations and up to 9 clusters for the dataset. Different symbols and line types encode different model parametrizations. The best model is taken to be the one with the highest BIC among the fitted models (black arrow).
