# Supplementary material for: Spatio-temporal migratory dynamics of Jasus frontalis (Milne Edwards, 1837) in Alexander Selkirk Island, Juan Fernández archipelago, Chile
Source: PLoS One. 2018 Jul 25;13(7):e0200146. doi: 10.1371/journal.pone.0200146 (PMC6059422; doi:10.1371/journal.pone.0200146)
Supplement: S2 Fig — Angles direction for each zone around AS Island in mark recapture periods A (M1R2), B (M2-R3) and C (M3-R3). Line around the circle represents the underlying circular distributions of displacement angles. (DOCX) [file pone.0200146.s004.docx]

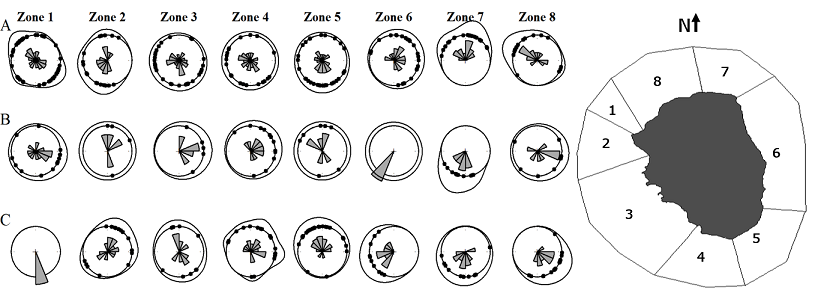


1. Angles direction for each zone around AS Island in mark recapture periods A (M1R2), B (M2-R3) and C (M3-R3). Lines around the circle represents the underlying circular distributions of displacement angles.
